# Does local endometrial injury in the nontransfer cycle improve the IVF-ET outcome in the subsequent cycle in patients with previous unsuccessful IVF? A randomized controlled pilot study

**DOI:** 10.4103/0974-1208.63116

**Published:** 2010

**Authors:** Sachin A Narvekar, Neelima Gupta, Nivedita Shetty, Anu Kottur, MS Srinivas, Kamini A Rao

**Affiliations:** Department of Reproductive Medicine, Bangalore Assisted Conception Center, #6/7 Kumara Krupa, High Grounds, Bangalore - 560001, India

**Keywords:** Endometrial injury, IVF/ICSI, Pipelle biopsy

## Abstract

**BACKGROUND:**

Management of repeated implantation failure despite transfer of good-quality embryos still remains a dilemma for ART specialists. Scrapping of endometrium in the nontransfer cycle has been shown to improve the pregnancy rate in the subsequent IVF/ET cycle in recent studies.

**AIM:**

The objective of this randomized controlled trial (RCT) was to determine whether endometrial injury caused by Pipelle sampling in the nontransfer cycle could improve the probability of pregnancy in the subsequent IVF cycle in patients who had previous failed IVF outcome.

**SETTING:**

Tertiary assisted conception center.

**DESIGN:**

Randomized controlled study.

**MATERIALS AND METHODS:**

100 eligible patients with previous failed IVF despite transfer of good-quality embryos were randomly allocated to the intervention group and control groups. In the intervention group, Pipelle endometrial sampling was done twice: One in the follicular phase and again in the luteal phase in the cycle preceding the embryo transfer cycle.

**OUTCOME MEASURE:**

The primary outcome measure was live birth rate. The secondary outcome measures were implantation and clinical pregnancy rates.

**RESULTS:**

The live birth rate was significantly higher in the intervention group compared to control group (22.4% and 9.8% *P* = 0.04). The clinical pregnancy rate in the intervention group was 32.7%, while that in the control group was 13.7%, which was also statistically significant (*P* = 0.01). The implantation rate was significantly higher in the intervention group as compared to controls (13.07% vs 7.1% *P* = 0.04).

**CONCLUSIONS:**

Endometrial injury in nontransfer cycle improves the live birth rate, clinical pregnancy and implantation rates in the subsequent IVF-ET cycle in patients with previous unsuccessful IVF cycles.

## INTRODUCTION

The success of *in vitro* fertilization and embryo transfer (IVF-ET) is contributed by a number of factors, which include the patient profile, uterine pathology, stimulation protocols, culture conditions, embryo quality and embryo transfer technique.

The process of implantation still remains a limiting factor in IVF-ET. For implantation to occur, a genetically normal blastocyst should hatch, appose, adhere, penetrate, and finally invade a well-synchronized endometrium, under the influence of estrogens and progesterone. Recently, a number of locally acting molecules including growth factors, cytokines, matrix metalloproteinases (MMPs), adhesion molecules, extracellular matrix components, and homeobox element containing genes, which mediate the action of the steroids hormones on the endometrium, have been discovered.[1,2]

The treatment of repeated implantation failure inspite of transfer of good-quality embryos continues to be a dilemma. Barash *et al*.[3] were the first to study the effect of endometrial injury on the pregnancy outcome. They demonstrated a significant doubling of the implantation, clinical pregnancy, and live birth rates in patients who underwent endometrial scraping in the cycle immediately preceding the IVF cycle. They hypothesized that the injury inflicted on the endometrium could lead to a massive secretion of growth factors and cytokines during the process of wound healing, which could help in implantation.

The objective of this RCT was to test the hypothesis that endometrial injury in the nontransfer cycle could improve the probability of pregnancy in the subsequent IVF cycle in patients who had previous failed IVF outcome.

## MATERIALS AND METHODS

### Study design and subject selection

This is a prospective, open-label, randomized controlled trial, involving patients undergoing IVF treatment at our center. The subjects were recruited from the period between May 2007 and July 2008. Approval for the study was obtained from the Institutional Review Board.

### Study participants

We enrolled patients undergoing fresh autologous IVF-ET, if they fulfilled all of the following inclusion criteria:

1. Patients with atleast one previous failed IVF-ET/ICSI cycles undergoing fresh autologous IVF/ICSI cycles.
2. Good responders in the previous IVF cycle.
3. Age: Less than or equal to 37 years.

We excluded patients with the following factors found to have a negative impact on implantation, namely:

1. Patients detected to have endometrial tuberculosis in the past, including those treated with antituberculous treatment.
2. Presence of intramural fibroid distorting the endometrial cavity/submucous myoma/ashermans syndrome.
3. Presence of sonographically detected hydrosalpinx.

We defined “good responders” as the patients who had developed at least four good-quality embryos (grade 1 and 2 of Veeck's grading) in the previous IVF cycles.

### Randomization

Patients found eligible for the study were offered to undergo endometrial sampling in the cycle prior to the embryo transfer cycle. After obtaining an informed consent, those willing to participate were randomized to either the intervention group or the control group at the time of hysteroscopy. The random allocation was based on computer-generated random numbers, sealed in consecutively numbered opaque envelopes, which were picked up by a nurse outside the operation theater. The study was not blinded, because the patients as well as the clinicians were aware of the treatment group.

### Treatment protocol

According to our internal protocol, all patients were evaluated with baseline day 3 FSH, antral follicle count, and a hysteroscopy on 7^th^ to 10^th^ day of the cycle prior to the embryo transfer cycle. Records of previous stimulation protocols and embryology details were reviewed.

The patients in the intervention group underwent endometrial sampling twice, with a biopsy catheter (Pipelle; Gynetics Medical Products, Hamont-Achel, Belgium), first on the day of hysteroscopy, and once again between 24^th^ to 25^th^ day of the nontransfer cycle on outpatient basis. After the introduction of the Pipelle into the uterine cavity, it was rotated 360 degrees and moved up and down four times after withdrawing the piston. All patients were prescribed Diclofenac 500 mg 30 minutes prior the procedure. Doxycyclin 100 mg was prescribed twice daily for 7 days after both the procedures. In order to avoid the possible confounding effect of antibiotic on IVF success, the control group was also prescribed Doxycyclin twice. Nonhormonal contraception was advised to the patients in both the groups in the nontransfer cycle.

Each woman recruited in the study underwent the same COH protocol that she had undergone in the previous IVF cycles, which included one of the three regimens, namely, long midluteal phase GnRH agonist suppression, GnRH antagonist or the GnRH agonist short protocol. In our unit, the protocols are selected by their primary physician depending on age, antral follicle count, and serum FSH levels. The GnRH agonists midluteal downregulation protocols are preferred for age groups ≤35 years, FSH <8 IU/l, and the combined antral follicle count ≥10. The short flare and antagonist protocols are preferred for age groups >35 years, FSH >8 IU/L, and antral follicle count <10. In the long protocol, patients were downregulated with 0.5 mg GnRH (Leupride® Sun Pharmaceuticals, Halol, Gujarat, India Ltd.) for the period of 10-14 days following which the dose was reduced to 0.2 mg and continued till hCG. After confirming adequate downregulation, FSH (Recagon® Organon, Ireland Ltd.), in the dose ranging from 150 to 250 IU, was commenced.

In the Antagonist group, flexible, multiple-dose regimens were used. GnRH antagonist (Orgalutran® Organon Ireland Ltd.) was started at a dose of 0.25 mg when at least one follicle reached 14 mm. Both Recagon® and Orgalutran® were continued till ovulation trigger.

The patients allocated to the short protocol were administered GnRH agonist 0.5 mg from day 2 onward and continued till the ovulation trigger. Gonadotropins were started from day 3 onward Subsequent monitoring was same as in the long protocol.

Women were scheduled for oocyte retrieval when at least three follicles reached a size of 18 mm. Oocyte retrieval was performed by the transvaginal route under ultrasound guidance, 35-hr after HCG trigger with 5000 IU, with the patient under conscious sedation. The morphology of each aspirated oocyte was noted after denudation with hyaluronidase. ICSI was performed for severe male factor, while combination of ICSI and conventional IVF was performed on some patients with unexplained infertility.

The embryos were classified according to Veeck's grading[4] as follows:

Grade 1 - preembryos with blastomeres of equal size and no cytoplasmic fragmentation;

Grade 2 - preembryos with blastomeres of equal size with cytoplasmic fragmentation equal to 15% of the total embryonic volume);

Grade 3 - uneven blastomeres with no fragmentation;

Grade 4 - uneven blastomeres with gross fragmentation (≥20% fragments).

Grade 1 or 2 embryos were considered to be good-quality embryos. Embryo transfer was performed with a Wallace® catheter (Smith Medical International Ltd., Hythe, Kent, UK) on day 3 a traumatically under ultrasound guidance by a senior consultant. In our center, we transfer upto three good-quality embryos in age group ≤35 years and up to four embryos in those above 35 years. Assisted hatching was not done in any of the patients. Luteal phase was supported with 600 mg/day of micronized progesterone vaginally till 12 weeks of pregnancy. β-hCG was determined 2 weeks after the embryo transfer. *P* values <0.05 were considered significant.

### Outcome variables

The primary outcome measure was live birth rate. The secondary outcome measures were implantation and clinical pregnancy rates. Live birth rate was calculated as the ratio of number of patients with live births divided by the number of patients who had embryo transfer.

Clinical pregnancy was defined as ultrasound evidence of fetal heart beat. Clinical pregnancy rate was calculated as the number of patients with clinical pregnancy divided by the number of patients who had embryo transfer. The implantation rate was defined as the number of gestational sacs as seen on transvaginal sonography divided by the number of embryos transferred.

### Statistical analyses

Statistical analyses were performed using the Statistical Package for the Social Sciences (10.0 SPSS Inc., Chicago). The Chi-square test was used for categorical variables and an independent sample *t*-test was used for continuous variables that were normally distributed. *P* value <0.05 was considered significant.

## RESULTS

**Figure 1:**
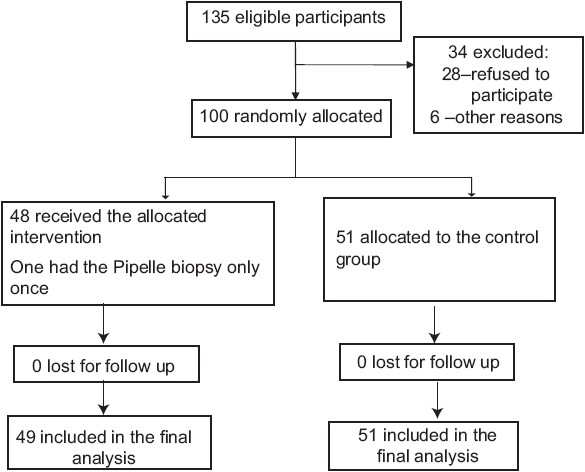
Participant flow

The participant flow is given in Figure 1. Out of a total of 135 patients found eligible for recruitment, 34 were excluded either due to refusal to participate (*n* = 28) or failure to meet the inclusion criteria (*n* = 6). Thus one hundred patients were randomized to the two groups with 49 women in the intervention group and 51 in the control group. One woman in the intervention group had Pipelle biopsy only once due to miscommunication. We performed an intention to treat analysis, and thus 49 women were analyzed in the intervention group and 50 in the control.

### Baseline characteristics

The baseline characteristics of patients and their outcome of controlled ovarian hyperstimulation are given in Table 1. All our patients had primary infertility. None of the patients were detected to have any pathology on hysteroscopy. There was no significant difference between the two groups as regards age, basal FSH, number of previous attempts, BMI, indication, number of preovulatory oocytes, the fertilization and cleavage rates, amount of recombinant FSH used, peak estradiol, and the number of good-quality embryos transferred. Although we did not perform a stratified randomization as regards the COH protocol, the distributions of patients in the three different protocols were not significantly different.

**Table 1:** Baseline characteristics and outcome of ovarian stimulation in the two groups

Variable	Intervention group	Controls group	*P* value
Age (years)	32.1 ± 3.4	32.3 ± 3.3	0.9
Basal FSH IU/L	5.01 ± 1.7	5.1 ± 1.7	0.9
Basal LH IU/L	4.1 ± 2.2	3.6 ± 1.7	0.2
Attempt	2.3 ± 0.52	2.5 ± 0.7	0.2
BMI	25.2 ± 2.8	25.9 ± 3.8	0.6
No. of preovulatory oocytes	5.73 ± 3.6	6.4 ± 4.3	0.9
Fertilization rate (%)	92 ± 13.2	96 ± 9	0.05
Cleavage rate (%)	93.3 ± 14.2	95.2 ± 13.3	0.4
Amount of recombinant	1491 ± 258	1479 ± 293	0.8
FSH used (IU)			
Peak estradiol level pg/ml	2072 ± 1401	2054 ± 1365	0.9
No. of good-quality embryos transferred	3.18 ± 0.8	3 ± 0.9	0.3
Total no. of embryos transferred	3.4 ± 0.5	3.3 ± 0.5	0.2
Mode of insemination (%)			
Conventional IVF	25 (51)	23 (45.1)	0.8
ICSI	6 (12.2)	6 (11.8)	
Combined IVF and ICSI	18 (36.7)	22 (43.1)	
Indication (%)			
Male	22/49 (44.9)	20/51 (39.2)	0.2
Tubal	6/49 (12.2)	11/51 (21.6)	
Endometriosis	2/49 (4.1)	4/51 (7.8)	
Unexplained	16/49 (32.7)	10/51 (19.6)	
Anovulation	3/49 (6.1)	3/51 (5.9)	
Combination	0	3/51 (5.9)	
Protocol (%)			
Long protocol	25/49 (51)	23/51 (45.1)	0.785
Short protocol	6/49 (12.2)	6/51 (11.8)	
Antagonist protocol	18/49 (36.7)	22/51 (43.1)	

All patients were monitored for the evidence of infection following the Pipelle biopsy. None of our patients developed infection. Also, other than spotting per-vaginuum for one to two days, there was no disturbance in the menstrual cycle.

### Outcome measures

The live birth rate was significantly higher in the intervention group compared to control group (22.4% and 9.8% *P* = 0.04) [Table 2]. The clinical pregnancy pregnancy rate in the intervention group was 32.7%, while that in the control group was 13.7%, which was statistically significant (*P* = 0.01). The implantation rate was significantly higher in the intervention group as compared to controls (13.07% vs 7.1%). There were two twins and one triplet pregnancy in the intervention group, while in the control group there was one twin and one triplet pregnancy. There were five spontaneous miscarriages, all after the appearance of fetal pole, in the intervention group, and two in the control group.

**Table 2:** Comparison of the outcome measures in the two groups

Parameter	Intervention group (%)	Control group (%)	*P* value*
Implantation rate	13.07	7.1	0.04
Clinical pregnancy rate	16/49 (32.7)	7/51 (13.7)	0.01
Live birth rate	11/49 (22.4)	5/51 (9.8)	0.04

* One sided *P* value was determined

## DISCUSSION

Barash *et al*. published a prospective case-control study of 45 “good responder” subjects who failed to conceive during one or more IVF-ET cycles. Endometrial samples were taken on days 8, 12, 21, and 26 of the menstrual cycle prior to their next IVF-ET. They reported a significantly doubled clinical pregnancy rate (66.7% vs 30.3%), implantation rate (22.7% vs 14.2%), and live birth rates (48.9% vs 22.5%).

Subsequently, Raziel *et al*.[5] reported a case control study in a group of 60 ICSI patients with higher order implantation failure (more than four unsuccessful embryo transfers of fresh embryos). The Pipelle biopsy was performed twice on days 21 and 26. They demonstrated an increased implantation (11% vs 4% *P* = 0.02), clinical pregnancy (30% vs 12% *P* = 0.02), and ongoing pregnancy rates (22% vs 8% *P* = 0.07) in the intervention groups.

### Our results concur with the above two studies

Our study was a randomized controlled trial unlike the other two studies. The other difference in the methodology was the number of times endometrial scrapping was performed in each patient.

Barash *et al*. performed endometrial biopsies four times in the spontaneous cycle (days 8, 12, 21, and 26), while Raziel el al. performed them twice on days 21 and 26. We felt it would be appropriate to perform this experimental procedure twice, once in follicular phase and once in luteal phase, mainly, to make it more acceptable to our patients.

The scientific explanation of the effect of endometrial injury is not yet fully clear. It was observed by Leob[6] in 1907 that scratching the progestational guineapig uterus resulted in decidualization of the endometrium. Later it was observed in rats that injection of oil also resulted in decidualization of progestational uterus.[7,8]

Zhou *et al*.[9] performed endometrial scratching, during controlled ovarian hyperstimulation (COH) in 60 patients treated with long protocol, who were good responders to previous hormonal stimulation and had an irregular endometrial echopattern on transvaginal sonography. They demonstrated a significantly increased implantation rate (33.3% vs 17.7% *P* < 0.05), clinical pregnancy rate (48.3% vs 27.8% *P* < 0.05), and live birth rates (41.6% vs 22.9% *P* < 0.05). In addition, 10 endometrial biopsy samples obtained on day 10 of the COH cycle were processed individually for gene chip hybridization. They found a total of 218 genes showing a statistically significant different expression when comparing the pregnant and nonpregnant patients. Of these, 41 were upregulated and 177 were downregulated. The genes for laminin alpha 4 and MMP1 were upregulated, while that of integrin alpha 6 were downregulated. While the exact function of laminin alpha 4 is not known, MMP1 and integrin alpha 6 play an important role in implantation.[10,11] The same authors also proposed that the injury-induced wound healing could result in the slowing of endometrial development, which could further lead to enhanced embryo-endometrial synchronization.

More recently, Kalma *et al*.[12] demonstrated that endometrial injury on days 11-13 and 21-24 of the nontransfer cycle resulted in upregulation of 183 genes and downregulation of 39 genes among those who had conceived. Most prominently upregulated genes was for the endometrial bladder transmembrane l protein (UPIb), which was found to be localized in the secretory vesicles of the glandular epithelial cells. Among the other genes, upregulated were genes for MUC1, crystallin alpha B, APOD, and PLA2. The authors hypothesized that the endometrial injury increases the expression of genes necessary for endometrial preparation for implantation.

Spandorfer *et al*.[13] studied the effect of autologous endometrial co-culture (AECC) and endometrial injury on pregnancy rate. Their results indicate that although AECC is a useful adjunct in multiple failed IVF attempts, there is no increase in the outcome when the biopsy is performed in the month prior to IVF cycle. This differed from our study as it was a case-control study and the biopsy was performed once, in the luteal phase.

There is a possibility that the diagnostic hysteroscopy could have caused mild endometrial injury in the control group as well. However, we believe that the injury induced by the Pipelle is deeper and secondly the injury inflicted in the luteal phase in the intervention group could have possibly resulted in the improvement in the pregnancy outcome.

The shortcoming of this trial is the small sample size and hence the power of the study as far as the live birth rate is concerned is 52%. A larger study needs to be done to verify the findings and improve the statistical power.

## CONCLUSION

Here we have demonstrated through a randomized control trial that the live birth rates clinical pregnancy and implantation rates significantly increase after endometrial scraping in the nontransfer cycle in patients with good-quality embryos. This phenomenon could be due to the injury-induced endometrial decidualization secondary to upregulation of genes encoding for locally acting mediators. Pipelle endometrial sampling is an easy and safe outpatient procedure. This certainly needs further investigation.
